# Thermal Decomposition and Thermal Reaction Process of PTFE/Al/MnO_2_ Fluorinated Thermite

**DOI:** 10.3390/ma11122451

**Published:** 2018-12-03

**Authors:** Jun Zhang, Junyi Huang, Xiang Fang, Yuchun Li, Zhongshen Yu, Zhenru Gao, Shuangzhang Wu, Li Yang, Jiaxiang Wu, Jiaying Kui

**Affiliations:** College of Field Engineering, PLA Army Engineering University, Nanjing 210001, China; zhangjun11067318@163.com (J.Z.); huangjunyi357@163.com (J.H.); 13390924860@163.com (Z.Y.); gygzr@sina.com (Z.G.); wushzh4394@126.com (S.W.); speedinessli@163.com (L.Y.); wujiaxiang1356@163.com (J.W.); xxkk6993@163.com (J.K.)

**Keywords:** fluorine-containing thermite, PTFE/Al/MnO_2_, thermal decomposition, TG/DSC-MS, XRD

## Abstract

To better understand the thermal decomposition and reaction process of a fluorine-containing powdery thermite, PTFE/Al/MnO_2_, reactions at different temperatures were investigated by the TG/DSC-MS technique. The corresponding reaction products were characterized with XRD phase analysis. Another three thermite materials, i.e., PTFE/Al, Al/MnO_2_, and PTFE/MnO_2_, were also prepared for comparison. Results showed that PTFE behaved as both oxidizer and reducer in PTFE/Al/MnO_2_ fluorinated thermite. The thermal decomposition and reaction process of as-fabricated ternary thermite could be divided into two stages—the mutual reaction between each of PTFE, Al, and MnO_2_ and the subsequent reaction produced between Al and Mn_2_O_3_/Mn_3_O_4_/MnF_2_. Compared with the three control systems, the specially designed ternary system possessed a shorter reaction time, a faster energy release rate, and a better heat release performance.

## 1. Introduction

Reactive materials can explode, deflagrate, and release a huge amount of chemical energy under impact. A typical representative of reactive materials [1], thermite features high energy, high density, flexible formulation, and a high adiabatic temperature. Conventional aluminothermic agents commonly comprise Al as the fuel and metal oxides (Fe_2_O_3_, MoO_3_, CuO, Bi_2_O_3_, MnO_2_, etc.) as the oxidant. However, the limited extent of a combination between an oxidant and a reductant can result in a low reaction rate, a smaller amount of actual heat release, an unconcentrated reaction process, a high initial temperature (>900 °C), a low energy release rate, and a compromised mechanical strength [2].

Research on fluorine-containing aluminothermic agents has received extensive attention recently. Due to a strong elemental electronegativity, fluorine atoms tend to lose the outermost electrons and become highly oxidizing. Polytetrafluoroethylene (PTFE) is a commercial fluorine-containing polymer [3,4,5,6] with a fluorine content of 76 wt.% [7]. For traditional oxide-based aluminothermic agents, Al and oxides react mostly in a condensed phase, whereas the addition of PTFE as a binder can trigger a redox reaction between PTFE and Al to yield AlF_3_. Studying the application of fluorinated polymers in thermite, Li [8] found that the low boiling point of AlF_3_ could prevent it from covering the Al particle surface and hindering the reaction. Puts and Crouse [9] investigated the effect of metal fluoride on PTFE decomposition, concluding that the catalytic effect of AlF_3_ could accelerate the reaction process and further promote the decomposition by inducing a 30 °C drop for the reaction temperature. In the meantime, the promotion effects exerted by PTFE were also reported for the thermal reaction, including a faster energy release, a higher flame temperature, and a greater reaction pressure of the thermite [10,11,12,13]. The addition of PTFE as a binder or matrix can magnify the original advantages, e.g., a high energy density and a high adiabatic temperature, and improve the mechanical strength of conventional powdered aluminothermic agents; the reaction materials will exhibit a better energy release as well. Furthermore, an as-fabricated damage element outperforms the inert one with its flexible formula, high oxygen carrying capacity, impact-based initiation, and its capability of generating a large number of elemental C (carbon) short circuit electronic components. At present, the research focus has been gradually shifting from thermal decomposition and reaction processes in traditional studies to the combustion performance of fluorine-containing thermites [5,14].

In this work, PTFE/Al/MnO_2_, a powdery fluorinated aluminothermic agent, was successfully fabricated by referring to the traditional aluminum heat agent and adding PTFE as a binder or matrix. Meanwhile, PTFE/Al, Al/MnO_2_, and PTFE/MnO_2_ were also prepared for comparison. Thermogravimetric (TG) analysis, differential scanning calorimetry (DSC), and mass spectrometry (MS) were used to analyze the reaction processes at different temperatures, while X-ray diffraction (XRD) was employed for the residue characterization. This study aims at providing practical guidance for the fluorination treatment of traditional thermites.

## 2. Experimental Section

### 2.1. Sample Preparation

PTFE (average particle size of 25 μm) was purchased from Shanghai 3F New Materials Co., Ltd. (Shanghai, China), Al powder (average particle size of 1–2 μm) was provided by Jintian Aluminum High-Tech Co., Ltd. (Luxi, Hunan, China) and MnO_2_ powder (average particle size of 3–5 μm) were commercially available at Europe Nano Technology Co., Ltd. (Shanghai, China). The formulations of the four materials prepared, i.e., PTFE/Al, Al/MnO_2_, PTFE/MnO_2_, and PTFE/Al/MnO_2_, are listed in Table 1.

The samples were prepared with the following procedure. First, the powder of various raw materials was added to a beaker at a designated ratio, followed by the addition of an appropriate amount of ethanol absolute. After the initial dispersion via 20 min stirring, the beaker was transferred into an ultrasonic system and kept for 30 min. The water bath was refreshed every 5 min during ultrasonication to prevent the solution from overheating and further triggering the reaction. The samples obtained were then dried in a vacuum oven at 60 °C for 48 h. Finally, the dried powder was sieved to produce uniform powdery materials.

### 2.2. Experimental Process

A combined testing system of thermogravimetry-differential scanning calorimetry (TG-DSC, NETZSCH-STA449C, NETZSCH, Bavaria, Germany) and mass spectrometry (MS, NETZSCH-QMS403C, NETZSCH, Bavaria, Germany) served as the primary analytical method for thermal decomposition and reaction processes of the four samples. Gas products were introduced into the mass spectrometer through a capillary tube, and their composition changes were monitored and recorded at different temperatures from 25 °C to 1000 °C with a fixed heating rate of 10 °C/min. To prevent air from participating in the reaction, experiments were carried out in a highly pure argon atmosphere with argon purging at 30 mL/min. Meanwhile, the capillary temperature was set at 200 °C to avoid gases cooling down within the capillary tube. The solid residues thermally analyzed at different temperatures were recovered and their phase composition was characterized with X-ray diffraction (XRD, Bruker D8 ADVANCE, Bruker, Berlin, Germany). The samples were scanned from 5° to 90° (2θ) at a scan step of 0.02°.

## 3. Results and Discussion

To develop a better understanding of the specific processes for the thermal decomposition and the reaction of PTFE/Al/MnO_2_ powdery thermite, the reaction processes of PTFE/Al, Al/MnO_2_, and PTFE/MnO_2_ at different temperatures should be analyzed in advance.

### 3.1. Thermal Decomposition and Thermal Reaction Processes of PTFE/Al

**Figure 1:**
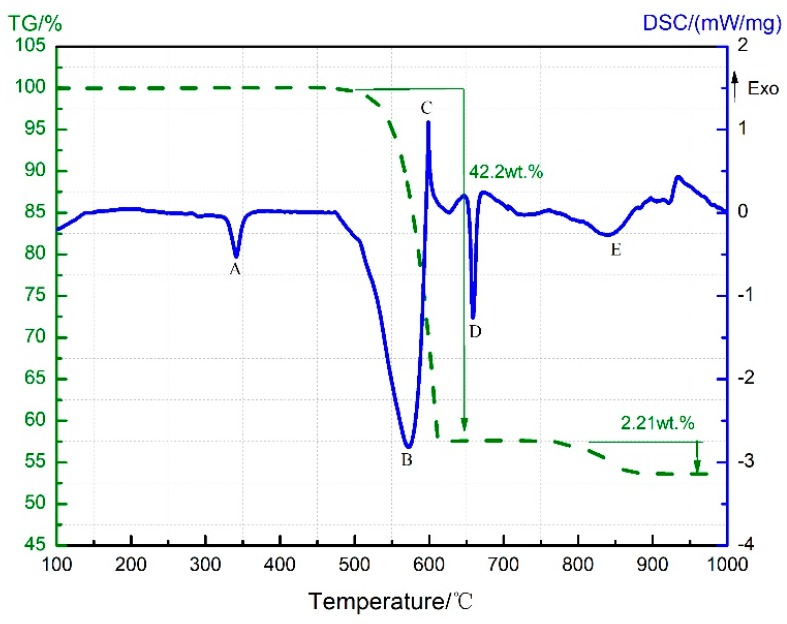
Thermogravimetry-differential scanning calorimetry (TG-DSC) curve of the PTFE/Al (polytetrafluoroethylene/aluminum) sample.

**Figure 2:**
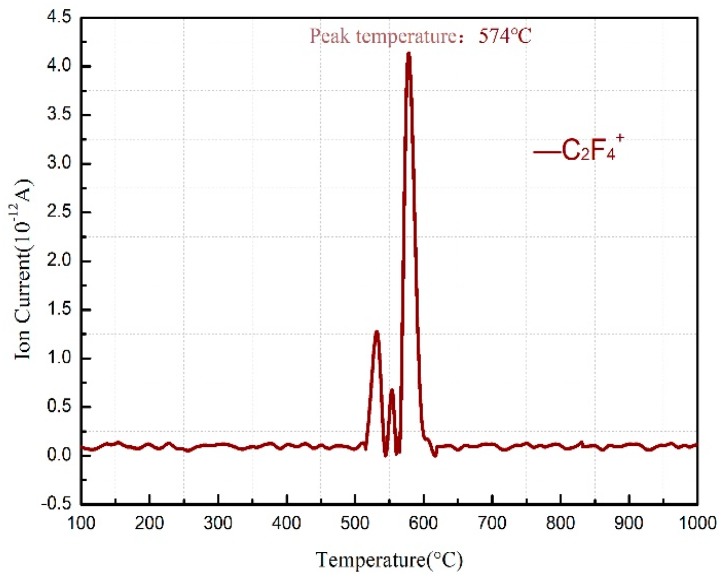
Mass spectrometry (MS) spectra of C_2_F_4_^+^ in PTFE/Al sample.

The TG-DSC curves recorded for the PTFE/Al thermal decomposition are depicted in Figure 1, in which a total of five peaks can be observed on the DSC curve. Peak A covers a temperature range from 323.2 °C to 358.1 °C, where no change shows up on the TG curve; it stands for the melting endotherm of PTFE (PTFE shows a melting point at 327 °C [15]). The TG curve suggests that the sample weight dropped sharply from 509 °C; meanwhile, C_2_F_4_^+^ ions (*m*/*z* = 100, Figure 2) were detected by mass spectrometry. Thus, peak B appearing then on the DSC curve should result from the endothermic decomposition of PTFE with C_2_F_4_ as the primary decomposition product. As for peak C, which begins at 597.9 °C and ends at 607 °C, it is attributed to the exothermic reaction between micron Al and PTFE decomposition products [3] that yielded AlF_3_ and C (carbon). Peaks B and C are actually the superimposed results of the endothermic PTFE decomposition and the exothermic PTFE/Al reaction. According to the MS spectrum presented in Figure 2, C_2_F_4_^+^ was produced and accumulated at 513 °C and then largely depleted due to its reaction with Al. However, since more AlF_3_ were produced, a mounting catalytic effect on PTFE decomposition [9] was exerted, which raised the C_2_F_4_^+^ content again to the peak value of 574 °C. As the PTFE decomposition ended at 619 °C, the content of C_2_F_4_ reduced gradually to zero (Figure 2). Furthermore, the excessive Al melted at about 660 °C, which gave rise to the endothermic peak D on the DSC curve.

**Figure 3:**
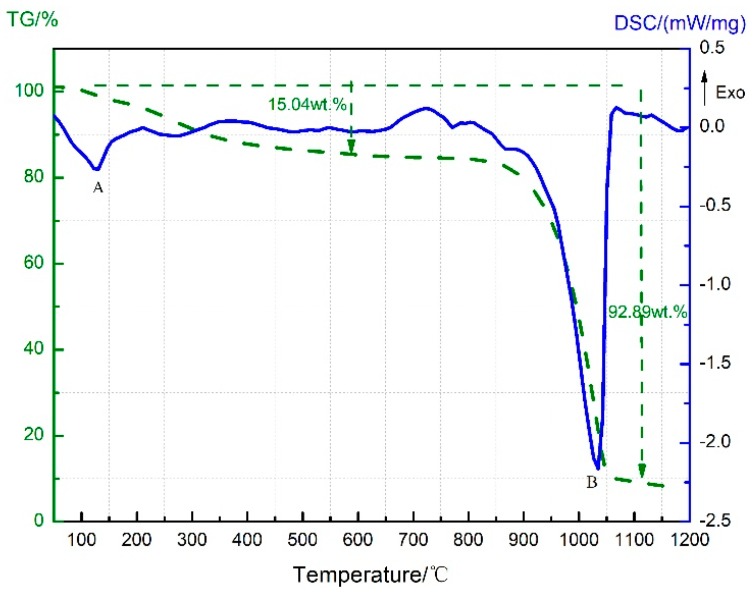
TG-DSC curve of neat AlF_3_.

At about 780 °C, the TG curve shows a second decline with a mass loss of 2.21 wt.%, and the endothermic peak E appears on the DSC curve correspondingly. The sample composition then was supposed to be C (carbon black), AlF_3_, and excess Al, and the endothermic sublimation of AlF_3_ might account for the appearance of peak E. The TG-DSC curve recorded for the heating of neat AlF_3_ up to 1200 °C under the same conditions is further provided in Figure 3 for verification. As can be seen, AlF_3_ sublimation gave rise to a remarkable drop at 832 °C on the TG curve, accompanied with an endothermic peak B on the DSC curve. Therefore, peak E in Figure 1 should also be attributed to AlF_3_ sublimation at high temperature. The only difference was that the sublimation temperature of AlF_3_ in the PTFE/Al sample was 50 °C lower than that of the neat AlF_3_.

**Figure 4:**
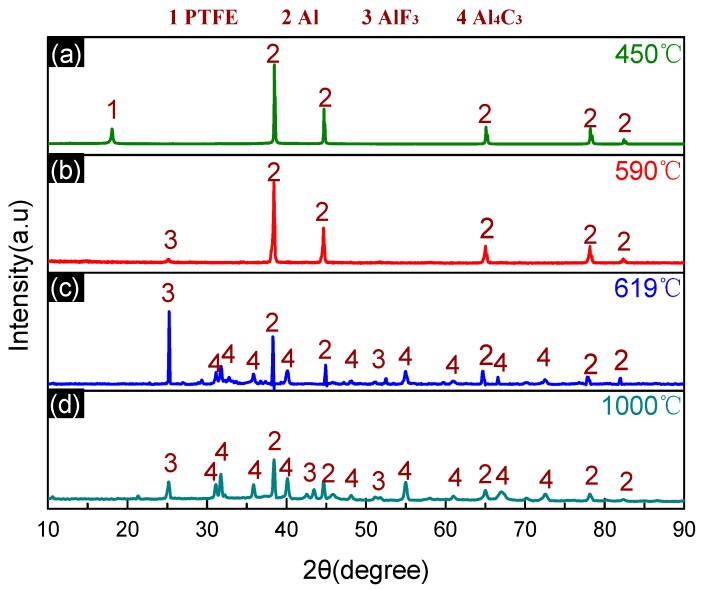
XRD patterns of PTFE/Al solid residues at different temperatures. (**a**) XRD patterns at 450 °C; (**b**) XRD patterns at 590 °C; (**c**) XRD patterns at 619 °C; (**d**) XRD patterns at 1000 °C.

The products of the PTFE/Al reaction at different temperatures were further characterized with the XRD phase analysis. The XRD patterns are depicted in Figure 4, while the product composition is listed in Table 2. The results indicate that Al_4_C_3_ was synthesized from C and excess Al at high temperatures.

### 3.2. Thermal Decomposition and Thermal Reaction Process of Al/MnO_2_

**Figure 5:**
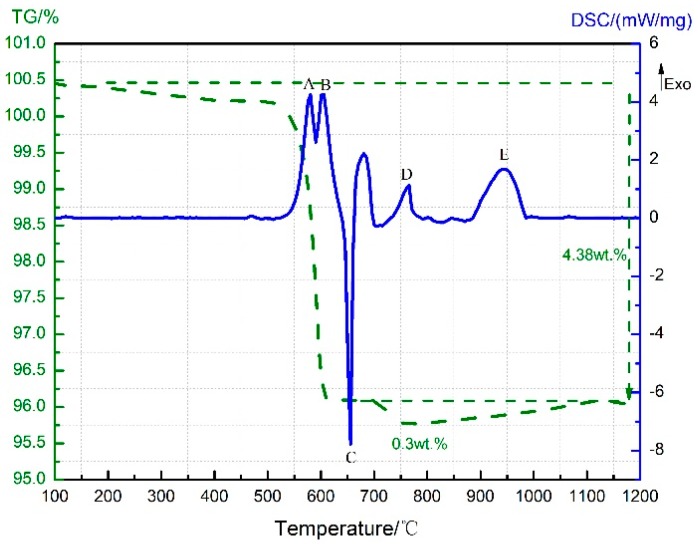
TG-DSC curve of Al/MnO_2_.

**Figure 6:**
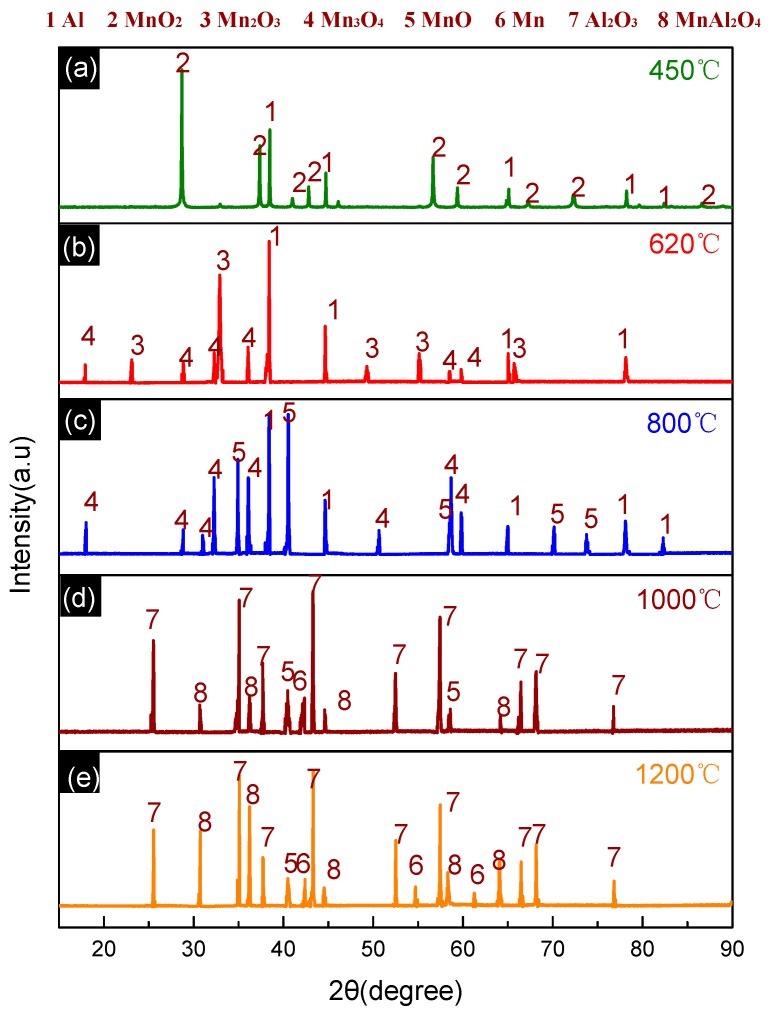
XRD patterns of Al/MnO_2_ solid residues at different temperatures. (**a**) XRD patterns at 450 °C; (**b**) XRD patterns at 620 °C; (**c**) XRD patterns at 800 °C; (**d**) XRD patterns at 1000 °C; (**e**) XRD patterns at 1200 °C.

Figure 5 presents the TG-DSC curves for the Al/MnO_2_ (30/70, *w*/*w*) thermal decomposition. MnO_2_ is chemically unstable and can decompose easily [16]. The two degradation stages on the TG curve correspond to the two-step decomposition process of MnO_2_ [17,18]. However, the appearance of two exothermic peaks, A and B, indicates that exothermic reactions still existed during the whole reaction process, and Figure 6b further suggests that the product consisted of Al, Mn_2_O_3_, and Mn_3_O_4_ after the reactions ending at 620 °C. Given that Mn_2_O_3_ decomposes into Mn_3_O_4_ at a temperature higher than 620 °C, the generation of Mn_3_O_4_ accompanied by heat release should be attributed to the reaction between Al and some MnO_2_—MnO_2_ has a stronger oxidizability than Mn_2_O_3_ and would react with Al preferentially. Al_2_O_3_, as a product, could not be detected by XRD due to its amorphous state or poor crystallinity [19]. Therefore, peaks A and B on the DSC curve were caused by the superposition of the Al/MnO_2_ reaction exotherm and the MnO_2_ decomposition endotherm. Moreover, peak C is assigned to the melting endotherm of excessive Al.

Peak D appearing later implies the existence of an exothermic reaction during the Mn_2_O_3_ decomposition, which ended as the temperature rose to about 800 °C. Meanwhile, the XRD patterns of the reaction products point out that the product components were Al, Mn_3_O_4_, and MnO, but without Mn_2_O_3_ (Figure 6). According to the process of MnO_2_ decomposition, MnO should not be included as a product. Hence, the reduction of the oxides of high-valence Mn by Al might account for the generation of MnO. A great amount of Al and Mn_3_O_4_ were left after the reaction was completed, which further proved that MnO was produced by Al reacting with Mn_2_O_3_.

Figure 6d is the pattern recorded at the end of the reaction represented by peak E (1000 °C), which suggests that Al_2_O_3_, Mn, MnAl_2_O_4_, and MnO existed as the product components while Al and Mn_3_O_4_ were not contained. As can be inferred, Mn and MnAl_2_O_4_ were formed during the exothermic reaction between Al and Mn_3_O_4_. Since Mn_3_O_4_ possesses a stronger oxidizability than MnO does, Al reacted with Mn_3_O_4_ preferentially. As for Al_2_O_3_, on the one hand, the reaction between MnO_2_ and Al would generate amorphous Al_2_O_3_, which further transformed into crystalline Al_2_O_3_ with a faster nucleation process at a higher temperature; on the other hand, the possible reaction between Al and Mn_3_O_4_ would also produce Al_2_O_3_ along with Mn.

### 3.3. Thermal Decomposition and Thermal Reaction Process of PTFE/MnO_2_

**Figure 7:**
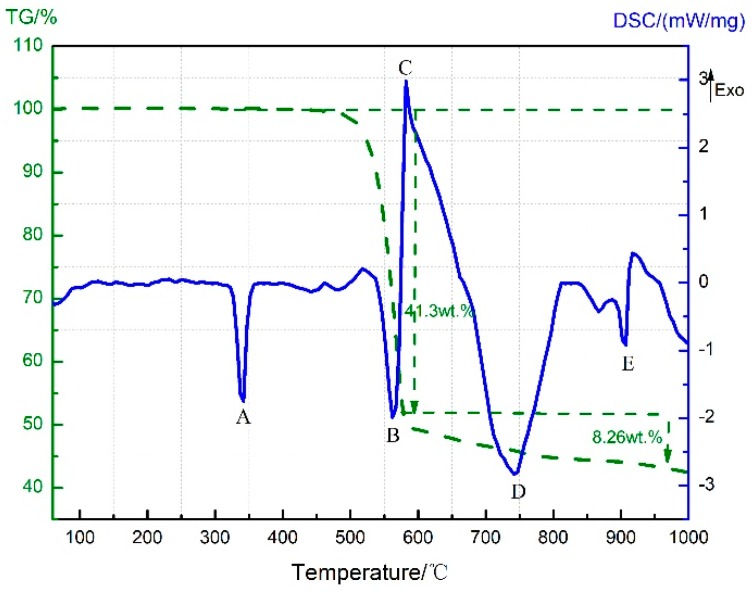
TG-DSC curve of PTFE/MnO_2_.

TG-DSC curves corresponding to the thermal decomposition of PTFE/MnO_2_ (42/58, *w*/*w*) are given in Figure 7. As discussed above in Section 3.1 and Section 3.2, peak A stands for the melting endotherm of PTFE, peak B for the decomposition endotherm of PTFE and MnO_2_, and peak D for the decomposition endotherm of Mn_2_O_3_.

The TG curve suggests a 41.3% decrease in the sample weight as the temperature rose from 470 °C to 580 °C. Theoretically, the weight loss should equal 47.34 wt.% if PTFE and MnO_2_ decomposed independently without any reaction happening between them. Thus, there might be an exothermic reaction between C_2_F_4_ and MnO_2_ (or Mn_2_O_3_), which gave rise to peak C on the DSC curve. The reaction mechanism is speculated as Formulas (1) and (2).

(1) $$C_{2}F_{4}+{2\mathrm{MnO}}_{2}\to {2\mathrm{CO}}_{2}+{2\mathrm{MnF}}_{2}$$

(2) $${2C}_{2}F_{4}+{2\mathrm{Mn}}_{2}O_{3}\to {4\mathrm{MnF}}_{2}+{3\mathrm{CO}}_{2}+C$$

**Figure 8:**
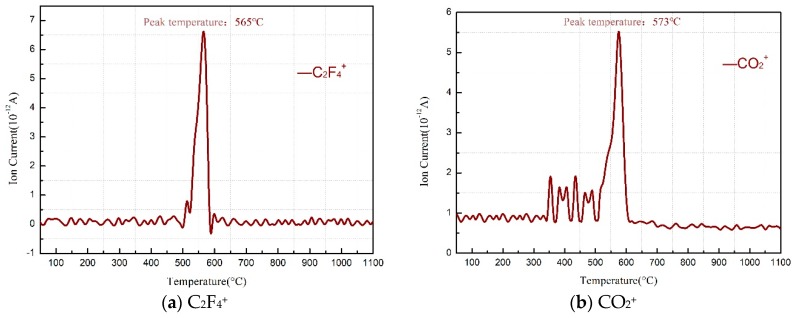
MS spectra of gaseous products from PTFE/Al/MnO_2_ samples. (**a**) MS spectra of C_2_F_4_^+^; (**b**) MS spectra of CO_2_^+^.

**Figure 9:**
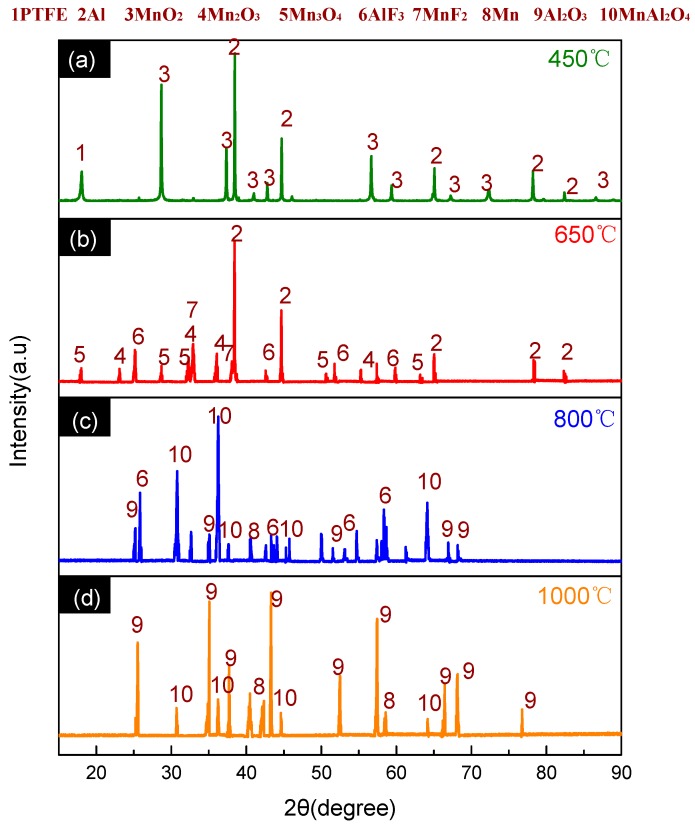
XRD patterns of PTFE/Al/MnO_2_ solid residues at different temperatures. (**a**) XRD patterns at 450 °C; (**b**) XRD patterns at 650 °C; (**c**) XRD patterns at 800 °C; (**d**) XRD patterns at 1000 °C.

During the reaction process of PTFE/Al/MnO_2_ samples, the amount of CO_2_ produced peaked at 573 °C (Figure 8a), and the formation of MnF_2_ was also detected (Figure 9b). With a slight endothermic peak (peak E of Figure 7) observed near 856 °C—the melting point of MnF_2_, the inclusion of CO_2_ and MnF_2_ in reaction products was thus confirmed. This further proves the occurrence of an exothermic reaction between PTFE and MnO_2_ (or Mn_2_O_3_). However, since this reaction coincided with the MnO_2_ decomposition, it is difficult to determine whether MnO_2_ or Mn_2_O_3_ was involved in the reaction.

The TG curve starts to decline sharply at 470 °C, while peak B on the DSC curve, which is an endothermic one, starts at 515 °C. As the decomposition temperatures of MnO_2_ and PTFE are around 530 °C and 514 °C, respectively, the weight loss between 470 °C and 515 °C should not be attributed to PTFE or MnO_2_ decomposition. Instead, it might result from the condensation reaction between PTFE and MnO_2_, as described by Formula (3).

(3) $${{(C}_{2}F_{4})}_{n}+{2\mathrm{nMnO}}_{2}\to {2\mathrm{nCO}}_{2}+{2\mathrm{nMnF}}_{2}$$

**Figure 10:**
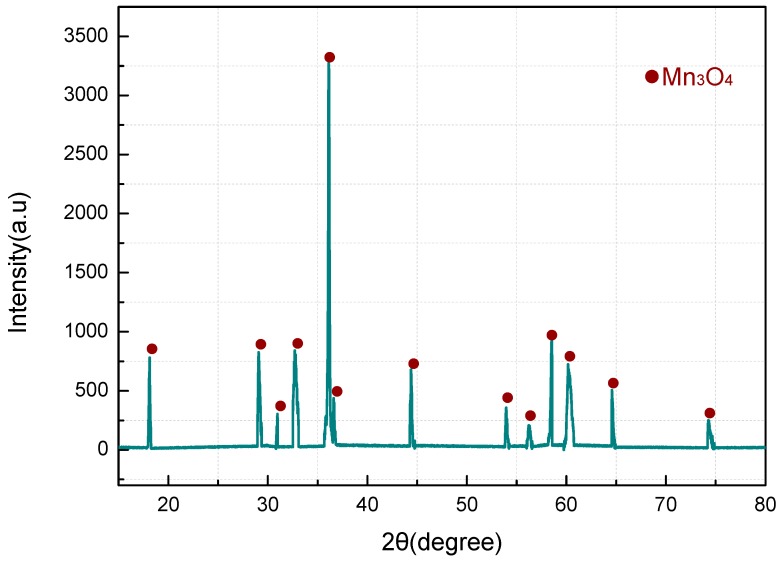
XRD map of PTFE/MnO_2_ after reaction.

At temperatures higher than 580 °C, both TG and DSC curves exhibit a downward trend. It is possible that the reaction between C_2_F_4_ and Mn_2_O_3_ pushed forward the decomposition of Mn_2_O_3_. Then, the solid residue after DSC analysis was subjected to XRD tests. Figure 10 shows the diffraction peaks only for Mn_3_O_4_, while no MnF_2_ has been detected. As merely about 5 mg of residue was left after DSC analysis, this ultralow content might account for MnF_2_ not being found.

### 3.4. Thermal Decomposition and Thermal Reaction Process of PTFE/Al/MnO_2_

**Figure 11:**
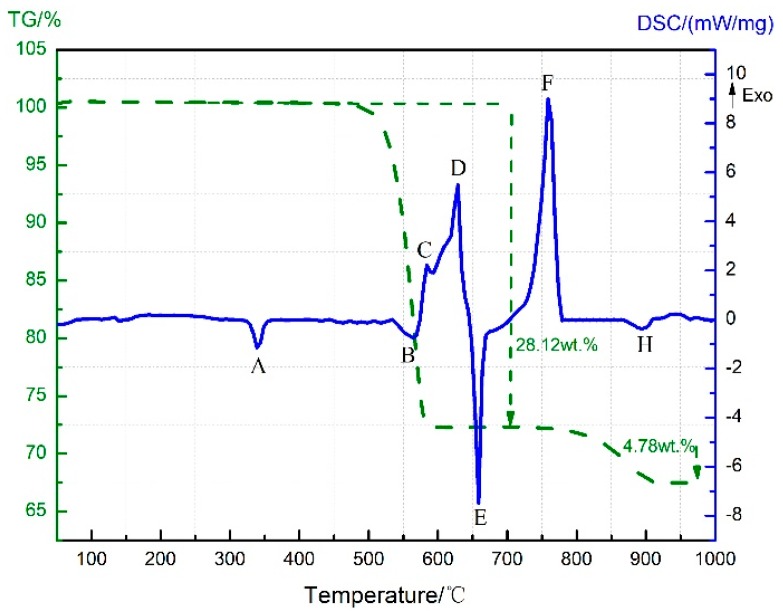
TG-DSC curve of PTFE/Al/MnO_2_.

Figure 11 demonstrates the TG-DSC curve for the PTFE/Al/MnO_2_ thermal decomposition. Same as that analyzed in Section 3.3, peaks A and B are endotherms due to PTFE melting and PTFE/MnO_2_ decomposition, respectively, while peak C results from the exothermic reaction between C_2_F_4_ and Mn oxides. Further, the mass spectrometric analysis of the gas evolution points out that CO_2_^+^ was formed at 475 °C without C_2_F_4_^+^ (Figure 8), so the weight loss suggested by the TG thermogram from 475 °C to 515 °C was indeed attributed to the condensation reaction of PTFE with MnO_2_.

The exothermic peak D covers a temperature range from 592 °C to 635 °C. As mentioned before, the exothermic reaction between Al and C_2_F_4_ in the PTFE/Al sample happened at 597–619 °C (Figure 1), and the one between Al and MnO_2_ in Al/MnO_2_ sample proceeded at 590–639 °C (Figure 5). Together with the XRD analyses in Figure 9b, which suggested that the reaction products of PTFE/Al/MnO_2_ sample comprised Al, Mn_2_O_3_, Mn_3_O_4_, MnF_2_, and AlF_3_ at 650 °C, peak D was considered to result from the superposition reactions of Al with C_2_F_4_ and MnO_2_.

Moreover, an exothermic peak F appears on the DSC curve from 714 °C (Figure 11), which is exactly the temperature range that corresponds to the exothermic peak D in Figure 5. Thus, there should be a certain correlation between these two exothermic reactions occurring individually in PTFE/Al/MnO_2_ and Al/MnO_2_. However, a drop on the TG curve for the Al/MnO_2_ sample is observed in the temperature interval that stands for Mn_2_O_3_ decomposition, while no apparent change can be detected for the PTFE/Al/MnO_2_ sample weight. Then, the XRD analysis of the corresponding reaction products was performed (Figure 9c), which demonstrates the presence of AlF_3_, Mn, Al_2_O_3_, and MnAl_2_O_4_ but the absence of Al, Mn_2_O_3_, Mn_3_O_4_, and MnF_2_. Therefore, peak F should be caused by the aluminothermic reaction between Al and Mn_2_O_3_/Mn_3_O_4_/MnF_2_. The newly generated products in this process were Mn, Al_2_O_3_, and MnAl_2_O_4_. This is consistent with the ones formed during the exothermic reaction of the Al/MnO_2_ sample between 880 °C and 987 °C. Besides, no more exothermic peaks can be found for the PTFE/Al/MnO_2_ sample in this temperature range, probably because the aluminothermic reaction before was much too intense and the enhanced exotherm reaction occurred in advance. Consequently, the exothermic reaction corresponding to peak F in the PTFE/Al/MnO_2_ sample equated to the combined reactions corresponding to peaks D and E in the Al/MnO_2_ samples. Furthermore, the TG curve shows no significant change between 714 °C and 783 °C. This may be owing to the limited generation of oxygen, as the fierce reaction made Mn_2_O_3_ reduced by Al once decomposed or even not decomposed yet.

The specific reaction process of the PTFE/Al/MnO_2_ sample between 714 °C and 783 °C could not be completely determined by simply analyzing the reaction products. Since both Mn_2_O_3_ and Mn_3_O_4_ may react with Al, MnO_x_ is used as an overall symbolization for Mn oxides (Mn_2_O_3_ and Mn_3_O_4_). The possible chemical reaction is described below:

(4) $$\mathrm{Al}+{\mathrm{MnO}}_{x}\to {\mathrm{Al}}_{2}O_{3}+{\mathrm{MnAl}}_{2}O_{4}+\mathrm{Mn}$$

Finally, the TG-DSC curves show a slight endothermic peak H between 870 °C and 910 °C accompanied with a decrease in the sample weight of 4.78 wt.%. Meanwhile, the XRD pattern in Figure 9d indicates Mn, Al_2_O_3_, and MnAl_2_O_4_ as the product components after the reaction but no AlF_3_. Therefore, the peak H corresponds to the sublimation endotherm of AlF_3_, which is consistent with the phenomenon observed in the PTFE/Al sample.

Energy release values during the exothermic reactions within various temperature ranges for the four samples, PTFE/Al, PTFE/MnO_2_, Al/MnO_2_, and PTFE/Al/MnO_2_, are gathered in Table 3.

As can be seen, the energy release in PTFE/Al and PTFE/MnO_2_ samples was only 20.32 J/g and 43.11 J/g due to the endothermic decomposition of PTFE, while the exotherm of the aluminothermic reaction between Al and MnO_2_ reached 94.41 J/g. The energy release value of the PTFE/Al/MnO_2_ sample achieved as high as 176.12 J/g, and the heat release was much higher than those of the first three. The mutual reactions between every two reactants in the PTFE/Al/MnO_2_ sample system exerted an overall promotion effect, so the energy release of PTFE/Al/MnO_2_ sample was greatly enhanced. For the exothermic reaction between 700 °C and 1000 °C, the PTFE/Al/MnO_2_ sample outperformed the Al/MnO_2_ sample with a greater heat release, an earlier triggered reaction, a shortened reaction time, and a faster energy release. Therefore, PTFE/Al/MnO_2_ exhibited heat release properties superior to those of PTFE/Al and Al/MnO_2_.

## 4. Conclusions

In this study, DSC/TG-MS and XRD techniques were employed to analyze the reaction process at different temperatures for PTFE/Al/MnO_2_ powdery fluorinated thermite. Meanwhile, the processes in PTFE/Al, PTFE/MnO_2_, and Al/MnO_2_ samples were also examined for comparison. The research can be concluded with the following results and findings.

1. PTFE oxidized Al into AlF_3_ in the PTFE/Al sample but were reduced into CO_2_ by Mn oxides (MnO_2_ or Mn_2_O_3_) in the PTFE/MnO_2_ sample. PTFE acted as both an oxidizer and a reducer in the PTFE/Al/MnO_2_ sample.
2. The reaction process of the PTFE/Al/MnO_2_ sample could be divided into two stages. In the first stage, PTFE and partial MnO_2_ decomposed. C_2_F_4_, the decomposition product of PTFE, could oxidize Al into AlF_3_ and get reduced into C. In addition, it would reduce Mn oxides (MnO_2_ or Mn_2_O_3_) into MnF_2_ and get oxidized into CO_2_. In the meantime, Al reacted with MnO_2_ to generate Mn_3_O_4_ and Al_2_O_3_. In the second stage, the constantly increasing temperature let excessive Al reduce Mn_2_O_3_/Mn_3_O_4_/MnF_2_ into MnAl_2_O_4_ and Mn, with AlF_3_ and Al_2_O_3_ produced simultaneously.
3. In the PTFE/Al/MnO_2_ sample, the mutual reaction between each two components could promote the overall reaction. Compared with the other three systems, the ternary system exhibited a shorter reaction time, a faster energy release, and a better exothermic performance.

## Figures and Tables

**Table 1:** Formulation of four experimental samples.

Sample	Composition (wt.%)		
	PTFE	Al	MnO_2_
Al/MnO_2_	\	30	70
PTFE/Al	51	49	\
PTFE/MnO_2_	42	\	58
PTFE/Al/MnO_2_	30	29	41

**Table 2:** Product composition in different temperature ranges during PTFE/Al (polytetrafluoroethylene/aluminum) thermal reactions.

Temperature/°C	<450	450–590	590–619	619–1000
Products	PTFE and Al	Al, AlF_3_, C, and C_2_F_4_ (g)	Al, AlF_3_, Al_4_C_3_, and C_2_F_4_ (g)	Al, AlF_3_, and Al_4_C_3_

**Table 3:** Energy release values during the exothermic reactions of the four samples.

Temperature Range	Energy Release (J/g)			
	PTFE/Al	PTFE/MnO_2_	Al/MnO_2_	PTFE/Al/MnO_2_
500–650 °C	20.32	43.11	94.41	176.12
700–1000 °C	\	\	129.06	209.14
